# Inosine monophosphate dehydrogenases are key players for functional development in Arabidopsis

**DOI:** 10.1111/tpj.71072

**Published:** 2026-08-04

**Authors:** Eva Dörfer, Zahra Sadeghi, Vincent Frick, Katharina Ebel, Adrian Heide, Tom Niehoff, Susanne Zehner, Lisa Fischer, Claus‐Peter Witte, Marco Herde, Torsten Möhlmann

**Affiliations:** ^1^ Plant Physiology, Faculty of Biology RPTU University of Kaiserslautern‐Landau Erwin‐Schrödinger‐Straße 67663 Kaiserslautern Germany; ^2^ Microbiology, Faculty of Biology RPTU University of Kaiserslautern‐Landau Erwin‐Schrödinger‐Straße 67663 Kaiserslautern Germany; ^3^ Molecular Nutrition and Biochemistry of Plants, Institute of Plant Nutrition Leibniz University Hannover Herrenhäuser Straße 2 30419 Hannover Germany

**Keywords:** metabolism, nucleotide, ribosome, photosynthesis

## Abstract

In plants, nucleotide *de novo* synthesis is required throughout development to provide building blocks for the synthesis of DNA, RNA and nucleotide‐derived cofactors. Inosine Monophosphate Dehydrogenases (IMPDH) are key enzymes in *de novo* guanylate synthesis. The genome of *Arabidopsis thaliana* encodes two IMPDHs and we identify IMPDH2 as the major isoform, being enzymatically active in the plant cytosol. Bimolecular fluorescence complementation (BIFC) analysis suggests that IMPDH1 and IMPDH2 interact with themselves and with Cytidine Triphosphate Synthetase (CTPS) isoforms, connecting purine and pyrimidine metabolism. Nucleotide quantification identifies IMPDH2 as a bottleneck in guanylate biosynthesis, and plants lacking this isoform suffer from nucleotide imbalance and limitation, causing reductions in ribosomal RNAs by reducing Target of Rapamycin (TOR) activity. Impaired photosynthesis and growth were further consequences thereof. In contrast, strong overexpression of IMPDH1 supported growth and led to altered organ development in 10% of corresponding plants presumably due to altered auxin metabolism during embryo development.

## Significance Statement

This study reveals that Inosine Monophosphate Dehydrogenase 2 (IMPDH2) is required for early Arabidopsis development and growth. Knock‐out mutants exhibit reduced amounts of metabolites (e.g. XMP, GMP, GTP) accompanied by reduced expression of genes in the GO‐Terms Ribosome and Photosynthesis at the seedling stage. This leads to reduced chlorophyll levels, impaired photosynthesis, and reduced growth. Marked overexpression of *IMPDH1* provoked abnormal organ development in 10% of cases presumably due to altered auxin metabolism.

## INTRODUCTION

Nucleotides are essential components of the genetic material and play important roles in cellular processes. They are involved in the storage and transmission of genetic information in the form of DNA and RNA. Further, nucleotides are needed as energy transmitters in many metabolic pathways and act as cofactors for sugar, starch, and phospholipid biosynthesis.

**Figure 1:**
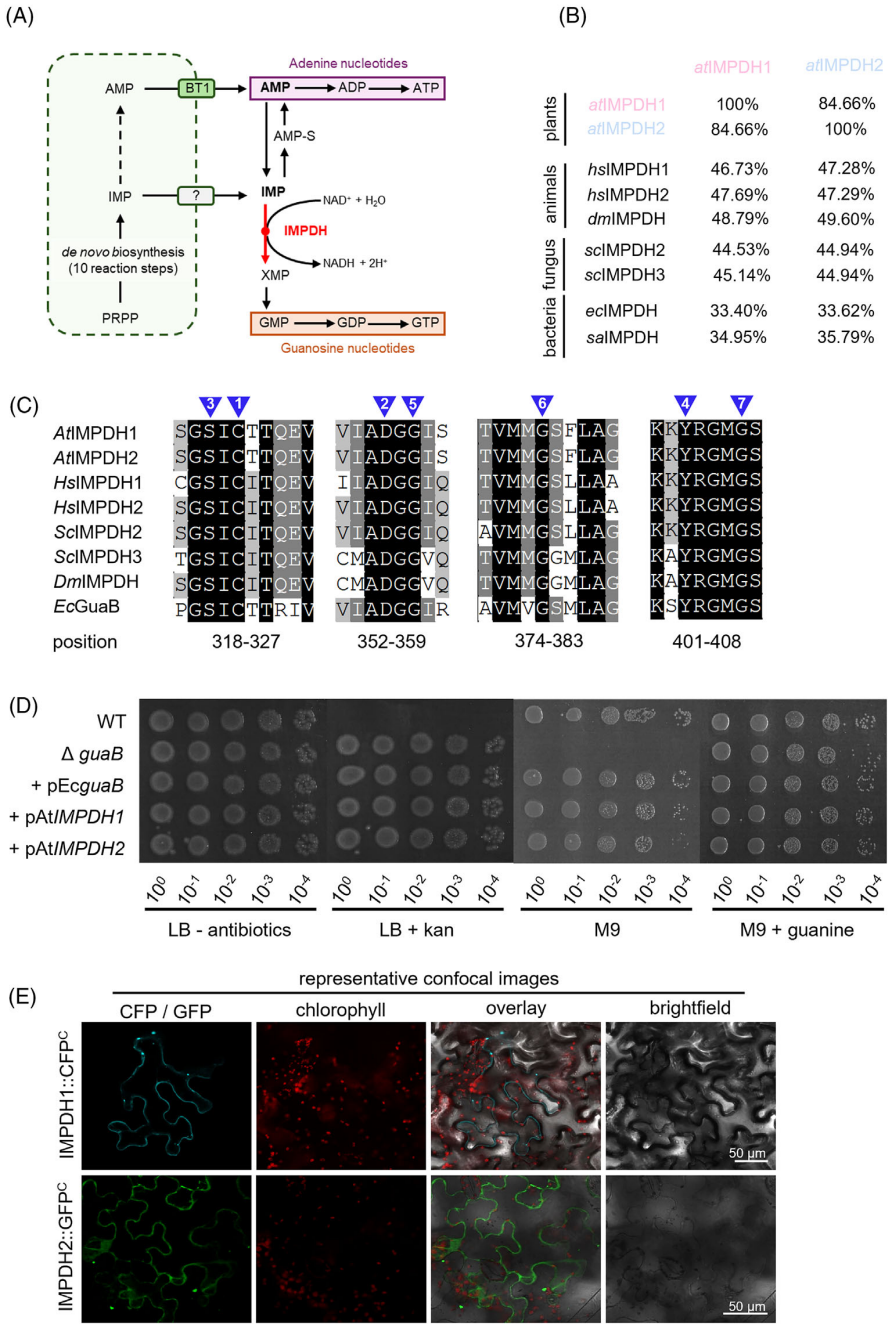
The enzyme IMPDH is responsible for the synthesis of XMP. (A) Scheme of purine *de novo* synthesis. IMPDH catalyzes reaction of IMP to XMP under consumption of NAD^+^ and H_2_O. (B) Homology of Arabidopsis IMPDH to corresponding proteins from selected animals, fungi and bacteria. Percentage of similar amino acid residues is given, based on protein alignments. at, *Arabidopsis thaliana*; hs, *Homo sapiens*; dm, *Drosophila melanogaster*; sc, *Saccharomyces cerevisae*; ec, *Escherichia coli*; sa, *Staphylococcus aureus*. (C) Partial alignments of selected IMPDH proteins taken from Figure S2, highlighting key conserved catalytic residues (blue arrow tips). (D) Spot test for AtIMPDH1 and 2 activity in complemented Δ*guaB E. coli* strains. (E) Localization of *IMPDH1::CFP* and *IMPDH2::GFP* transiently expressed in *Nicotiana benthamiana*.

Plant purine *de novo* synthesis consists of 10 reaction steps in the plastid to generate inosine monophosphate (IMP). Two further plastidic reactions lead from IMP to AMP, while GMP is synthesized from IMP in the cytosol (Zrenner et al., 2006). Inosine Monophosphate Dehydrogenase (IMPDH) catalyzes the oxidation of inosine monophosphate (IMP) to xanthosine monophosphate (XMP) in the presence of NAD^+^. XMP is then aminated by Guanosine 5′‐Monophosphate Synthetase (GMPS) using the amide group of glutamine as nitrogen donor and ATP for energetic coupling resulting in the production of GMP as well as glutamate, AMP, and pyrophosphate as side products (Figure 1). In a similar type of reaction, the final step in pyrimidine *de novo* synthesis is catalyzed by CTP Synthetase (CTPS) performing the amination of uridine triphosphate (UTP) to cytidine triphosphate (CTP). There are five *CTPS* genes in Arabidopsis and the corresponding enzymes are all located in the cytosol (Daumann et al., 2018; Moffatt & Ashihara, 2002; Witz et al., 2012; Zrenner et al., 2006). The synthesis of CTP by CTPS relies heavily on GTP as an allosteric activator which connects purine and pyrimidine biosynthesis (Chang & Carman, 2008; Daumann et al., 2018; Levitzki & Koshland Jr., 1972). Both IMPDH and CTPS can form filamentous structures named ‘cytoophidia’ in vertebrates. Under certain conditions, mixed cytoophidia were observed, which apparently contain CTPS and IMPDH protein. However, by high resolution microscopy it was found that these mixed cytoophidia were formed by two or more filaments lying closely together separated by a tiny gap, whereby each filament either consisted of CTPS or IMPDH alone (Chang et al., 2018; Keppeke et al., 2015).

Human IMPDH exists as two isoforms (Carr et al., 1993) and mutations in both can lead to diseases. Therefore, vital interest in IMPDH biology results from the observation of cell proliferation and tumor growth in humans correlating with increased purine *de novo* synthesis and specifically IMPDH activity, making IMPDH a target for cancer therapy (Jackson et al., 1975). The retina is characterized by a high demand for purine nucleotides and mutations in human IMPDH1 cause retinal degeneration (Bowne et al., 2002). Mutations in human IMPDH2, however, have been associated with a neurodevelopmental disease (Burrell & Kollman, 2022). Thus, both human isoforms exhibit unique but different physiological functions.

Although IMPDH also exists in two isoforms in many animals and plants, Drosophila (*Drosophila melanogaster*) only harbors one isoform (Sifri et al., 1994) and the same holds true for plants such as Rice (*Oryza sativa*), Barley (*Hordeum vulgare*), Potato (*Solanum tuberosum*) and Wine (*Vitis vinifera*), to name a few (TAIR, The Arabidopsis Information Resource, www.arabidopsis.org). Apparently, physiological diversification of IMPDH is not required *per se*.

In its monomeric form, IMPDH has two domains, a catalytic domain which binds the substrates IMP and NAD^+^, and a regulatory domain which can bind adenine and guanine nucleotides at distinct sites, thereby altering the oligomeric state and activity of IMPDH (Burrell & Kollman, 2022). Assembly of IMPDH cytoophidia reflects an upregulation of IMPDH activity correlating with rapid cell proliferation. From this, it was concluded that maintenance of the GTP pools requires IMPDH filamentation (Keppeke et al., 2018).

In plants, purine *de novo* synthesis proceeds in plastids and results in the export of AMP by the BT1 transporter. It has been speculated that inosine monophosphate (IMP) can be directly transported into the cytosol by a yet unknown transporter (Leroch et al., 2005; Witte & Herde, 2020) since the IMPDH and GMPS reactions for GMP synthesis are located in the cytosol. IMPDH from plant tissue has first been described and purified from soybean (Cao & Schubert, 2001), and two putative IMPDH isoforms have been identified by homology in Arabidopsis, IMPDH1 (At1g79470) and IMPDH2 (At1g16350), both sharing 84% identity at amino acid level with another (Collart et al., 1996; Maekawa et al., 2024; Witte & Herde, 2020).

The aim of this work was to perform a detailed characterization of plant IMPDHs exploiting the potential of the model plant *Arabidopsis thaliana*. Furthermore, as Arabidopsis harbors two IMPDH isoforms, we aimed to uncover which individual roles they might have. As IMPDH from eukaryotes can assemble into filaments, approaches to reveal whether this can happen with Arabidopsis IMPDH, too, were undertaken. By establishing knock‐out and overexpression mutants for both *IMPDH* isoforms, a major role of IMPDH2 during ribosome biogenesis and the establishment of photosynthesis was revealed, while IMPDH1 has a role in functional embryo development and cotyledon formation. Furthermore, support for IMPDH1‐IMPDH2 interactions was gathered by bimolecular fluorescence complementation (BIFC) experiments, as well as for interactions between IMPDH and CTPS proteins. In addition, subcellular (co‐)localization studies by fluorescent fusion proteins were performed. Transcriptomic analysis reveals alterations in gene expression in four‐ and eight‐day‐old *IMPDH2* knock‐out lines and was complemented by nucleotide metabolite quantification in seedlings that were obtained from imbibed seeds transferred to growth conditions for 12 h, 48 h, or for 8d.

## RESULTS

### Phylogenetic relations and sequence conservation among IMPDH family members

The central position of IMPDH in purine metabolism suggests a vital role of this enzyme in the establishment of purine nucleotide homeostasis (Figure 1A). The high similarity between both Arabidopsis isoforms of 85% suggests a redundant function in XMP synthesis. Similarities to fungal and animal homologs are in the range between 44.5 and 49.6% and thus still high, whereas the similarity of *At*IMPDH to bacterial IMPDH ranges between 33.40 and 35.79% (Figure 1B). Phylogenetic analysis reveals clustering of IMPDH isoforms within Brassicaceae, however in all other plant species analyzed, the isoforms 1 and 2 are more closely related to each other compared to any IMPDH from another species (Figure S1) also supporting the idea, that Arabidopsis has two IMPDH isoforms because they have different, for example tissue specific functionalities. Some plants like *Manihot esculenta*, *Nicotiana tabacum*, *O. sativa* only harbor a single IMPDH. In animals with mostly two isoforms, IMPDH1 and IMPDH2 form distinct clusters (Figure S1). Furthermore, algal and fungal IMPDHs are more closely related to the plant cluster than bacterial IMPDHs. (Figure S1). A protein alignment of IMPDH from Arabidopsis, Human, Drosophila, Yeast and *Escherichia coli* IMPDHs reveals conservation of the substrate binding sites. The catalytic residue Cys319/322 (Hedstrom/AtIMPDH1 Figure 1C and Figure S2, blue triangel #1) is completely conserved, as are most of the residues that interact with IMP (Figure S2; Figure 1C). These residues include Asp 358/355, #2, which forms hydrogen bonds to the ribose hydroxyls of IMP. Ser317/320, #3 and Tyr405/403, #4, which forms hydrogen bonds to the phosphate via their hydroxyl groups, are also completely conserved. Gly360/357, #5 and Gly381/378, #6, which interact with the phosphate via main chain NHs and GLY409/407, #7, which forms a hydrogen bond to the purine ring via its NH, are also invariant (Hedstrom, 2009). In contrast, residues relevant for allosteric regulation within the Bateman domain exhibit substantially lower overall conservation.

In IMPDH, filament formation is driven by the stacking of functional tetramers into octamers, which then polymerize. Key residues mediating these interfaces include Tyr12, Arg356, and Ser275. Mutating these sites in human IMPDH2 (e.g., Y12A or R356A) disrupts polymerization, while variants like S275L induce constitutive filament formation (Anthony et al., 2017). Whereas Y12 is only conserved among human isoforms in our alignment, R356 is conserved among human IMPDH but exchanged to Q in all other sequences except for *E. coli*. S275 is found invariant in all sequences analyzed (Figure S2, green triangels).

### Arabidopsis IMPDH1 and IMPDH2 are enzymatically active

Enzymatic activity of IMPDH1 and IMPDH2 was assessed in an *E. coli* complementation approach using a mutant of the *E. coli* IMPDH encoded by *guaB*. A Δ*guaB* knock‐out strain (JW5401‐KC, Keio Collection) is unable to grow on minimal medium because GMP *de novo* synthesis is compromised. This mutant was complemented with either *guaB* from *E. coli* (Δ*guaB* + pEc*guaB*), or with the codon optimized *IMPDH1* gene from *A. thaliana* (Δ*guaB +* pAtIMPDH1opt) or IMPDH2 (Δ*guaB +* pAtIMPDH2opt). For genotyping of the corresponding *E. coli* strains see Figure S3. All tested strains grew on control M9 minimal medium with 0.5 mM guanine which can be salvaged to GMP. However, on M9 medium without guanine the Δ*guaB* strain failed to grow. This defect could be complemented by *guaB* (strain Δ*guaB* + pEc*guaB*) and importantly also by *IMPDH1 and 2* (strain Δ*guaB +* pAtIMPDH1opt.;Δ*guaB +* pAtIMPDH2opt) revealing that both *At*IMPDH isoforms are enzymatically functional (Figure 1D).

### Both IMPDH isoforms locate to the cytosol and can interact with themselves and with cytidine triphosphate synthase (CTPS)

AMP and presumably also IMP are exported from plastids for the further synthesis of adenylates and guanylates (Leroch et al., 2005; Witte & Herde, 2020; Zrenner et al., 2006). As IMPDH isoforms do not carry N‐terminal extensions that could serve as organellar targeting sequences, it is likely that they are located in the cytosol. To test this assumption, IMPDH1 and IMPDH2 were synthesized as fusion proteins with C‐terminal Cyan Fluorescent Protein (CFP) or Green Fluorescent Protein (GFP), respectively, using transient expression in *Nicotiana benthamiana*. CFP and GFP signals were observed at the cell margin in epidermal leaf cells, which is indicative of a cytosolic localization because the cytosol surrounds the extensive central vacuole in these cells. Often, fluorescence appeared in macromolecular structures, as punctae or oval clusters (Figure 1D; Figure S4).

**Figure 2:**
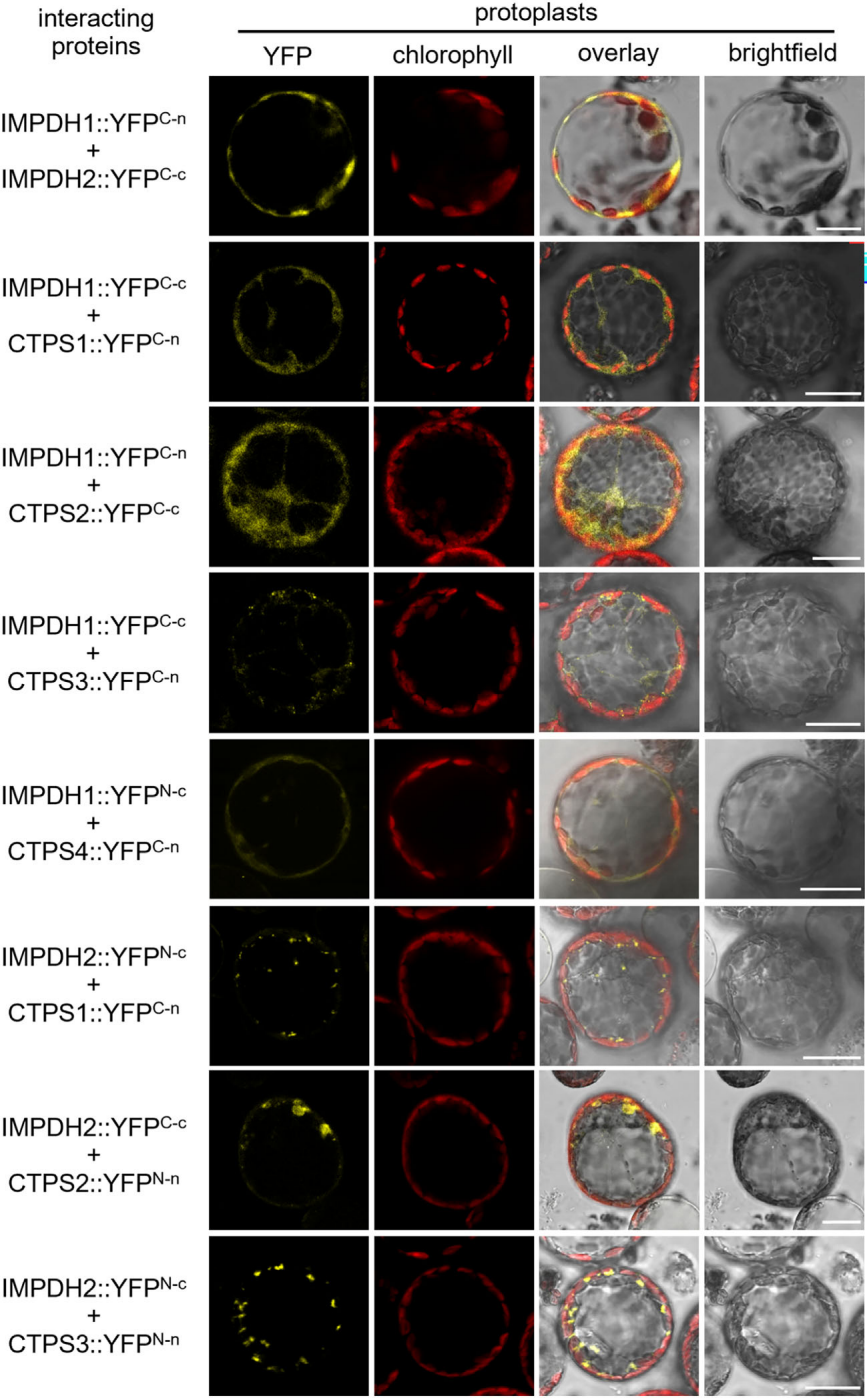
Bimolecular Fluorescence complementation (BiFC) of IMPDH isoforms and CTPS isoforms. Shown are interaction of combinations of each two different isoforms after transient expression in *Nicotiana benthamiana* leaves. IMPDH and CTPS proteins were fused C‐ or N‐terminally with either a C‐ or N‐terminal YFP fragment, as indicated. Confocal laser scanning microscopy was applied for imaging. Scale bar = 20 μm.

IMPDH and CTPS catalyze key reactions in the synthesis of the pyrimidine ribonucleotides GTP and CTP. For this, CTPS enzymes from animals and plants require allosteric activation by GTP (Daumann et al., 2018; Lunn et al., 2008), which functionally links purine and pyrimidine *de novo* synthesis. Furthermore, both proteins can form filaments which can closely align, suggesting a functional interaction (Chang et al., 2018). As indications for filament formation of plant CTPS isoforms were reported as well (Alamdari et al., 2021; Daumann et al., 2018; Krämer et al., 2022), we were curious about interactions between IMPDH isoforms themselves and with CTPS variants. In an initial approach, YFP N‐and C‐terminally halves were translationally fused to IMPDH and CTPS N‐ and C‐termini for bimolecular fluorescence complementation (BiFC) analysis. Dihydroorotase (DHO), a cytosolic enzyme of pyrimidine de novo synthesis, was used as a negative control, as to our knowledge no protein–protein interaction with this enzyme has been reported (Moffatt & Ashihara, 2002; Witz et al., 2012). We observed that IMPDH1 interacts with IMPDH2 and all four tested CTPS isoforms. IMPDH2, on the contrary, only interacts with CTPS1–3 (Figure 2). In the case of IMPDH1, only the interaction with CTPS3 results in the formation of punctae and clusters, whereas the IMPDH2 interaction with CTPS1, 2, and 3 promoted the formation of such structures. Interactions between IMPDH2 and CTPS4 or of both IMPDHs with DHO have not been observed (Figure S5).

To obtain clues about the functional state of the observed structures, *IMPDH2* was transiently expressed in tobacco leaves. Transfected leaf discs were incubated with various metabolites. These were the substrate of IMPDH, IMP, the IMPDH‐specific inhibitor mycophenolic acid (MPA), ATP, which produces an active conformation of IMPDH in humans, and GTP, which triggers feedback inhibition in humans (Buey et al., 2015; Johnson & Kollman, 2020), and GTP together with ATP. Under control conditions, as observed before, only a few IMPDH2 clusters were monitored. Neither IMP, MPA, nor GTP addition triggered polymer formation. Interestingly, when ATP was added, many polymers could be observed, and the enzyme was no longer present in dissolved form. When GTP and ATP were supplemented simultaneously, the ATP‐induced polymer formation was noticeable, with IMPDH2 also partially returning to a more dissolved state (Figure S5).

### IMPDH2 mutants show severe developmental defects

To obtain insights into the physiological function of Arabidopsis IMPDH isoforms, T‐DNA mutants for both were identified. These are the homozygous mutants of *IMPDH1* (SAIL_5_B10C; *impdh1‐1*) and *IMPDH2* (SALK_008653; *impdh2‐0* and SALK_201269; *impdh2‐1*). Corresponding transcript levels of the mutated *IMPDH* gene were not detected, while transcripts of the respective other isoform were reduced as well to about 50% of wild‐type level (Figure S7A–C). Moreover, complementation lines for *impdh1‐1* and overexpression lines for both IMPDH isoforms were generated. Three *IMPDH1* complementing lines (*IMPDH1* c#1–3) with transcript levels close to WT were identified (Figure S7D). Furthermore, three *IMPDH1* overexpression lines (*IMPDH1* ox#1–3) with medium to high overexpression (10‐, 21‐, and 46‐fold relative to WT) were generated. For *IMPDH2*, three lines (*IMPDH2* ox#1–3) with medium to high overexpression (4‐, 14‐, and 33‐fold relative to WT) were obtained (Figure S7E,F).

All mutant plant lines analyzed in this work were able to complete a full lifecycle. Due to the high similarity of both IMPDH proteins we speculated about a functional redundancy. Crosses between *impdh1‐1* and *impdh2‐0* resulted in 10.5% wildtypes, 31.6% heterozygous (het) plants for both alleles and 42.1% homozygous (hz) *IMPDH2* mutants (Table S1). No homozygous double knock‐out plants could be observed, indicating that the homozygous loss of both *IMPDH* isoforms is embryo lethal, as previously described (Maekawa et al., 2024). In line with this, aborted seeds in developing siliques were already observed in both single *IMPDH* knock‐out mutants at rates of 27.3% for *impdh1‐1* and 25.4% for *impdh2‐0*. Seed abortion was accompanied by reduced length of siliques from both knock‐out lines (Figure S7G,H).

**Figure 3:**
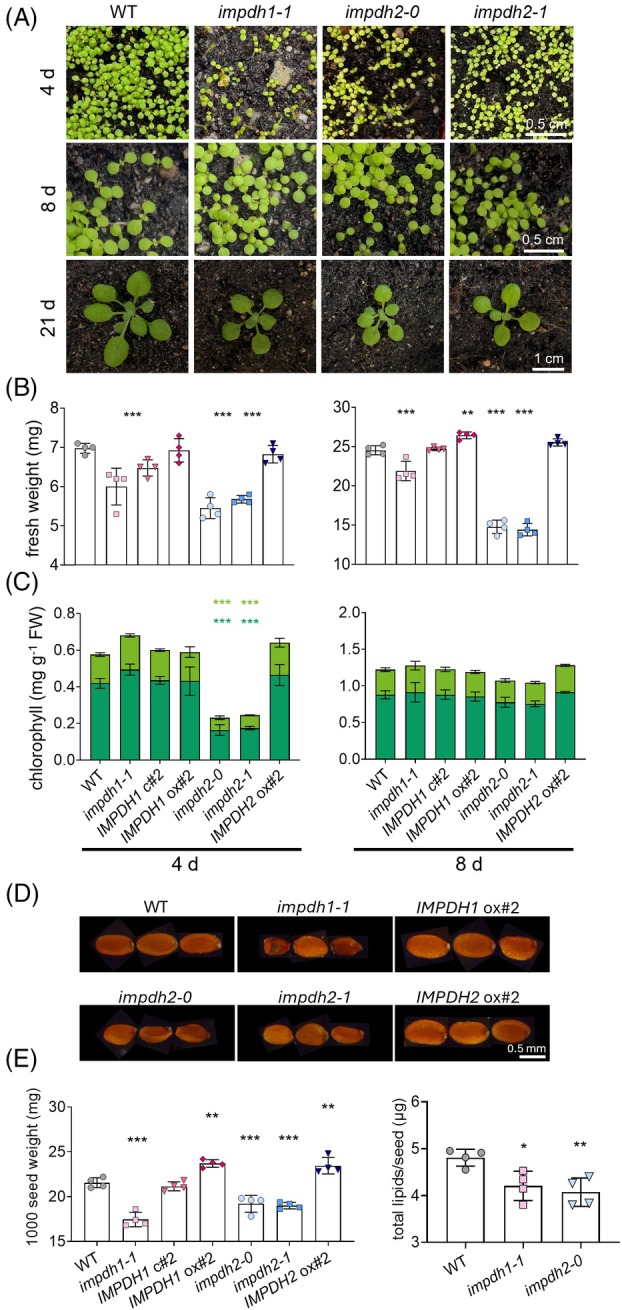
Phenotypical analysis of *IMPDH* loss‐of‐function mutants. (A) Phenotypic analysis of WT, *impdh1‐1*, *impdh2‐0* and *impdh2‐1* Arabidopsis plants. Plants were grown under standard conditions (21°C day and night temperature, 10 h day length and 120 μE light intensity) for different length of time as indicated. (B) Fresh weights of WT, *impdh1‐1*, *IMPDH1* c#2, *IMPDH1* ox#2, *impdh2‐0*, *impdh2‐1* and *IMPDH2 ox#2* after 4 and 8 days of growth. (C) Chlorophyll a and b levels of the same plants as in (B) after 4 and 8 days of growth. (D) Typical appearance of mature seeds from *IMPDH* mutants. (E) 1000 seed weight and total lipids per seed of WT and *IMPDH* mutants. Error bars represent mean values ± SD. Asterisks indicate significant differences between WT and mutant using a one‐way ANOVA followed by a Dunnett's multiple comparisons test: **P‐value ≤0.01, ***P‐value ≤0.001.

In *impdh1‐1*, and in both *impdh2* mutant lines, reduced fresh weight (FW) levels were seen, accompanied by severe chlorosis in both *impdh2* lines after 4 days of growth on soil (Figure 3A). While plant size was still reduced after 8 days of growth, the leaves of *impdh2* lines were not chlorotic anymore. After 21 days, *impdh1‐1*, *impdh2‐0* and *impdh2‐1* still showed a reduced rosette size (Figure 3A). The differences in plant size throughout growth were also resembled in the corresponding FW. During 4–21 days of growth, all *impdh* ko lines had a lower FW compared to WT. While 4‐day‐old *impdh1‐1* showed 14% lower FW compared to WT, in *impdh2‐0* and *2‐1* the reduction was 22 and 19%, respectively. After 8 days of growth FW reductions were 11% for *impdh1‐1* and app. 40% for both *IMPDH2* mutant lines (Figure 3B). Opposite to this, the *IMPDH1*ox#2 mutants were larger after 8 days of growth (Figure S7A) and had a slightly higher FW (8%, Figure 3B). The chlorotic *impdh2‐0* and *impdh2‐1* contain only approximately one third of chlorophyll compared to the WT after 4 days of growth. These deficits adjust to the WT chlorophyll level after 8 days (Figure 3C).

Mature seeds of both *IMPDH* knock‐out mutant lines were smaller and showed deformations compared to WT seeds. Furthermore, the seed weight of *impdh1‐1* and *impdh2‐0* was lower compared to WT seeds, whereas the corresponding overexpression seeds were heavier and larger (Figure 3D,E). As Arabidopsis seeds contain up to 46% lipids (Hölzl & Dörmann, 2019), the total lipid content was quantified. We observed reduced total lipid contents based on seed weight for *impdh1‐1* and *impdh2‐0* (Figure 3F).

**Figure 4:**
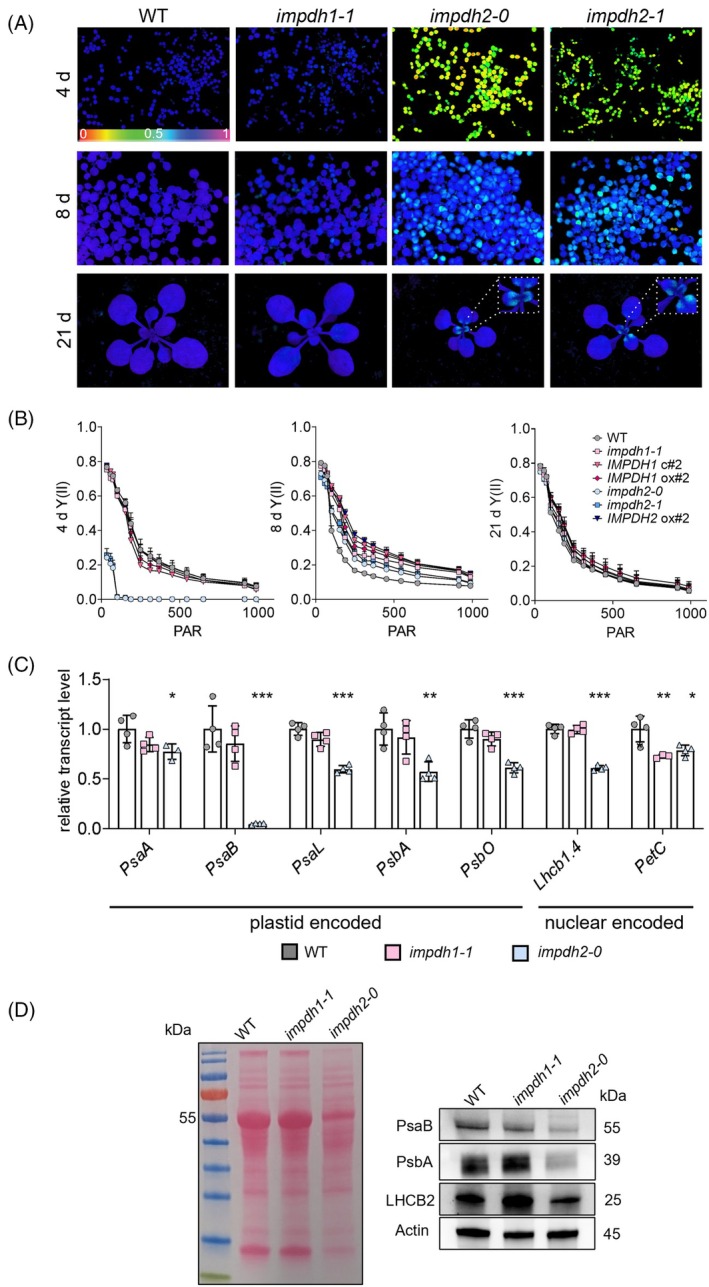
Photosynthetic alterations in *impdh2* mutants. (A) Representative PAM images of WT, *impdh1*, *impdh2‐0* and *impdh2‐1* are depicted for maximum PSII capacity (Fv/Fm). (B) Light curves of Y(II) for WT and *IMPDH* mutants after 4, 8 and 21 days of growth. (C) Expression levels of photosynthetic genes in *impdh1‐1* and *impdh2‐0* after 4 days of growth. (D) Ponceau staining of total extracted protein corresponding to 4 mg FW per genotype (4‐day‐old seedlings). Immunoblot of photosynthesis associated proteins in 4‐day‐old seedlings with Actin as loading control. Error bars represent ± SD. Asterisks indicate significant differences between WT and mutant using one‐way ANOVA followed by Dunnett's multiple comparisons test: *P‐value ≤0.05, **P‐value ≤0.01, ***P‐value ≤0.001.

As chlorosis was observed in *IMPDH* mutants which is an indicator for impaired photosynthetic effectiveness, we performed pulse amplitude modulation (PAM) measurements. Here, we could detect a massive decrease of PS yield (Fv/Fm) at 4 days of growth for *impdh2‐0* and *impdh2‐1*. After 8 days Fv/Fm was still slightly reduced in these seedlings. After 21 days of growth, Fv/Fm reduction was restricted to the newly developed leaves in both *impdh2* mutants, indicating a vital function of IMPDH2 during cell proliferation as proliferating tissues have a high demand for nucleotide supply (Figure 4A,B; Figure S8). Additionally, transcript levels of photosynthesis‐associated genes were reduced in 4‐day‐old *impdh2‐0* seedlings (Figure 4C). Especially *psaB* was massively reduced in transcript amount. When inspecting protein contents of *IMPDH* mutants, it appeared that in *impdh2‐0* seedlings a lower extractable amount of protein was apparent and reduced RUBISCO levels largely contributed to this (Figure 4D). Steady state levels of photosynthesis proteins (PsaB, PsbA, and LHCB2) were reduced as well, in line with the observed reductions in corresponding transcript levels (Figure 4D).

### Morphological alterations in *IMPDH1* over expressors may result from altered auxin metabolism

**Figure 5:**
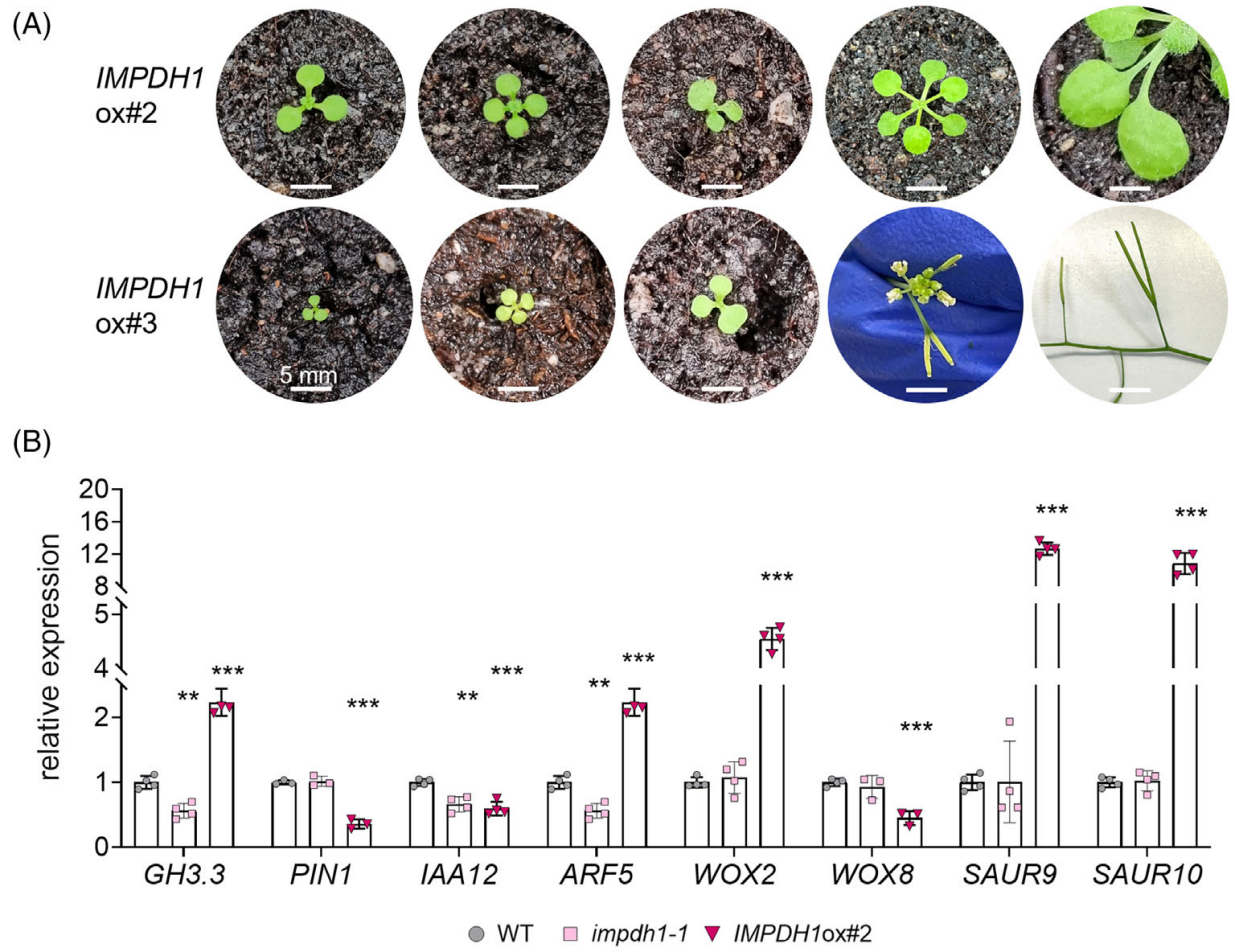
Phenotypical analysis of *IMPDH1* overexpression mutants. (A) Phenotypic analysis of *IMPDH1* ox#2 and ox#3 Arabidopsis plants showing alterations in cotyledon number and shape as well as altered silique growth. (B) Gene expression levels of auxin‐related genes in WT, *impdh1‐1* and *IMPDH1* ox#2 embryos harvested 6 days after fertilization (DAF). Error bars represent ± SD. Asterisks indicate significant differences between WT and mutant using a one‐way ANOVA followed by a Dunnett's multiple comparisons test: *P‐value ≤0.05, **P‐value ≤0.01, ***P‐value ≤0.001.

In the strong overexpression lines *IMPDH1 ox#2* and ox#3, besides slight growth improvements, morphological alterations were observed. About 10% of *IMPDH1* ox#2 and *IMPDH1* ox#3 plants showed abnormal leaf numbers. At the seedling stage, apparently, 3 or 4 cotyledons were observed, and these plants then also grew 3 or 4 new leaves at a time (Figure 5A). Growth of one petiole that divides into leaves was seen in these plants as well. Similar effects were seen for siliques, where two siliques grow from the same pedicel (Figure 5A). Additional cotyledons and alterations were also observed in mutants for PIN1 and PINOID proteins involved in auxin metabolism (Furutani et al., 2004). Therefore, we speculated that imbalanced auxin metabolism during embryo development might account for the observed improper organ development. Levels of auxin responsive genes in embryos 6 DAF were quantified, and it appeared that transcripts of *SAUR9* and *SAUR10* (small auxin responsive 9 and 10) were markedly upregulated in *IMPDH1* ox#2 (Figure 5C). This increase in *SAUR* transcripts indicates massive overproduction of auxin in the *IMPDH1 ox#2* embryos. *GH3.3*, on the other hand, is an auxin conjugation enzyme that breaks down excess auxin. The increased expression of *GH3.3* confirms the accumulation of auxin in the *IMPDH1 ox#2* embryos, whereas the reduced transcript content of *GH3.3* in *impdh1‐1* embryos indicates a reduced amount of auxin. The expressions of the transcription factor ‘Auxin‐Responsive Factor 5’ (ARF5) and the auxin repressor IAA12 also show typical responses to increased auxin levels in the *IMPDH1 ox#2* embryos. ARF5 activates auxin signaling and developmental genes. *IAA12* binds to *ARF5*, thereby blocking it (Hamann et al., 2002). However, increased auxin levels lead to the repression of *IAA12*, so the regulation of auxin‐responsive genes by *ARF5* can take place again (Maraschin Fdos et al., 2009). The transcript levels of the genes *PIN1*, *Wuschel‐Related‐Homeobox 2* (*WOX2*), and *WOX8* were examined to obtain information on the distribution of the phytohormone. The reduction in *PIN1* expression in the *IMPDH1* ox#2 embryos indicates reduced auxin transport in the basal part of the embryo. This is consistent with the increase of *WOX2* expression, which is in the apical part of the embryo, and decreased *WOX8* expression in the *IMPDH1* ox#2 embryos, which is needed for cell fate determination in the basal part of the embryo. Taken together, these auxin imbalances in the embryo likely contribute to altered organ development.

### Nucleotide metabolism is altered in *IMPDH* mutants

As we speculated about limiting guanylate supply as reason for the low chlorophyll content and the reduced FW in *IMPDH2* knock‐out mutants, a rescue experiment was performed. For this, WT, *impdh2‐0*, and *IMPDH2* ox#2 seedlings were grown in liquid culture with GMP, guanine, or mycophenolic acid (MPA), an IMPDH‐specific inhibitor. With GMP supplementation, *impdh2‐0* seedlings lose their chlorotic phenotype and chlorophyll levels resemble those of the WT, whereas the supplementation with guanine leads to an increase in FW in *impdh2‐0* to WT level under standard conditions (Figure S9A). MPA dependent reduction of chlorophyll levels was less pronounced in *IMPDH2* ox#2 compared to WT (Figure S9A), supporting our initial hypothesis that IMPDH2 activity impacts the establishment of functional chloroplasts. Histological staining of *proIMPDH2*::GUS lines revealed staining of cotyledons, shoot apical meristem, and roots in 6‐day‐old seedlings. When incubated with guanine or GMP, staining of cotyledons appeared slightly reduced. In contrast, when the IMPDH inhibitor MPA was applied, *proIMPDH2::GUS* staining clearly increased. Thus, *IMPDH2* expression seems to respond inversely with respect to guanylate availability (Figure S9B).

**Figure 6:**
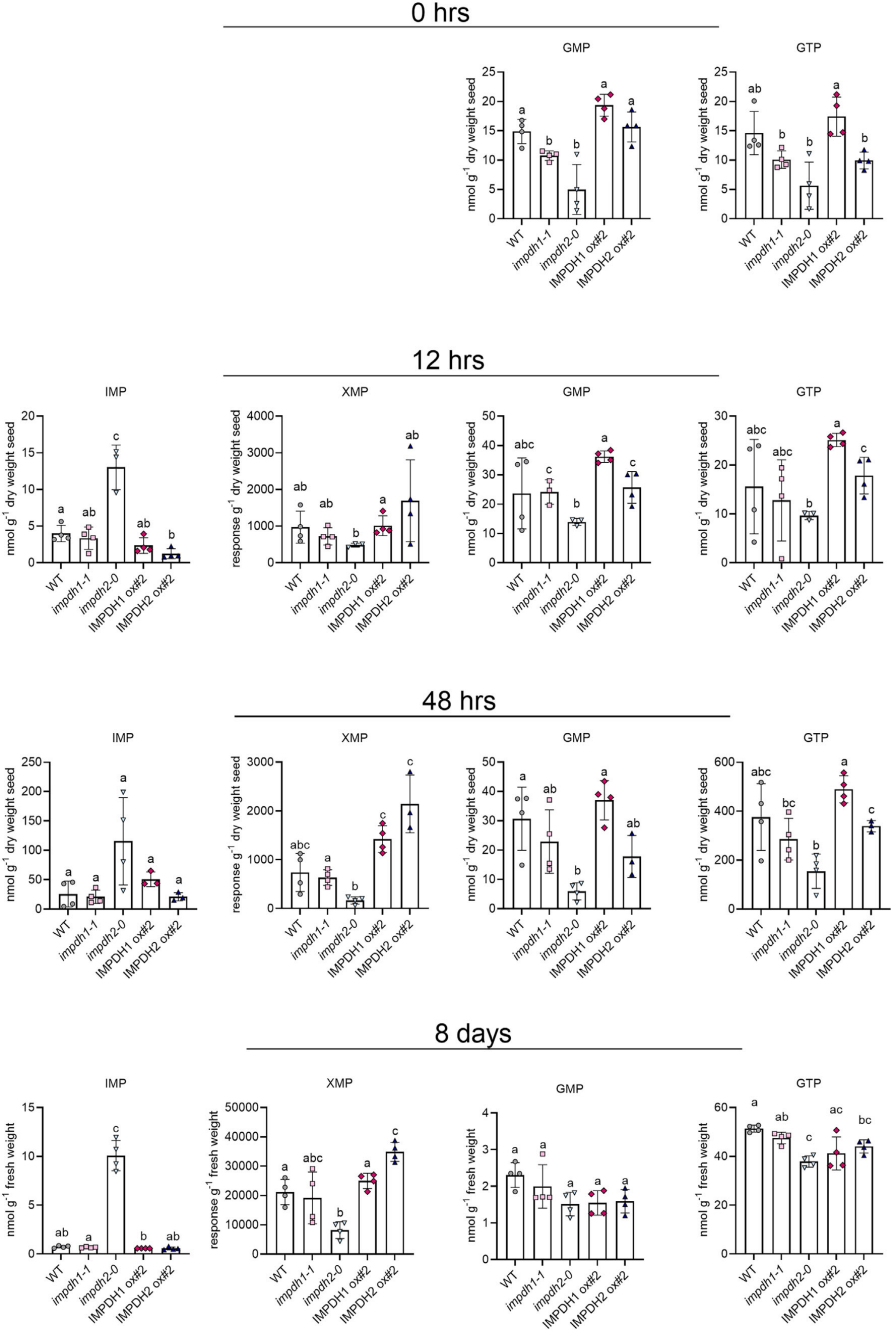
Metabolic analysis of guanylate biosynthesis pathway nucleotides in genetic *IMPDH* variants over a developmental time course. Nucleotides quantified by LC–MS after imbibition (0 h) and after 12 h, 48 h and 8 days of growth. Three or four biological replicates were analyzed, error bars are SD. The statistical analysis used the two‐sided Tukey's multiple pairwise comparison test with the sandwich variance estimator. Different letters indicate *P* values <0.05.

Due to IMPDH's central role in guanylate metabolism, corresponding mutants were subjected to metabolite analysis by LC–MS. For this, different timepoints during germination were sampled at the end of the imbibition period at 4°C and after 12 h, 48 h, and 8 days of growth. Directly after imbibition, neither IMP nor XMP were detectable, but the content GMP was reduced strongly in *impdh2‐0* seeds and also slightly in *impdh1‐1* seeds, whereas this was not the case in the overexpression lines (Figure 6). In principle, the same was observed for GTP but with higher statistical uncertainty (*P* > 0.05). At the later time points, IMP and XMP became detectable in seedlings. The IMP content was generally higher and the XMP content lower in *impdh2* seedlings, while this was not the case in the *impdh1‐1* line. Similarly, GMP and GTP concentrations were only reduced in the *impdh2‐0* line. The *IMPDH2* ox#2 complemented the metabolic phenotype of the corresponding mutant at all time points, sometimes even overcompensating the mutant by reducing the IMP content below wild‐type level (at 12 h) and increasing the XMP concentration above that of the wild type (at 8 d and in tendency, but with *P* > 0.05, also at 48 h). The metabolite content of *IMPDH1* ox#2 and the wild type did not differ at any time point. Interestingly, the metabolic pattern for GMP and GTP in the different *IMPDH* variants was in principle mirrored by the other nucleotides (AMP and ATP, UMP and UTP, CMP and CTP, Figure S10). The statistical support for differences in individual metabolites is often weak, but overall, the nucleotides in the different *IMPDH* variants show similar trends.

The metabolite data indicate that IMPDH2 is the more important enzyme overall, which agrees with its generally higher expression compared to IMPDH1. However, in the imbibed seed, IMPDH1 clearly contributes to the overall IMP to XMP turnover. Changes in the guanylate content by alteration of IMPDH activity results in parallel changes of other nucleotides presumably by regulation of nucleotide homeostasis that has also been observed in other cases (Straube et al., 2021).

To reveal the consequences of nucleotide limitation on changes in gene expression in *impdh2* mutants, an RNA‐Seq analysis was performed. In this analysis, *impdh2‐0* seedlings were compared to WT controls of the same size early after germination. In one comparison, 4‐day‐old *impdh2‐0* seedlings (showing chlorosis) were compared to 3‐day‐old WT seedlings. Furthermore, slightly older seedlings (*impdh2‐0* 8 day‐old and WT 6‐day‐old) were compared. Here the *impdh2‐0* seedlings had already overcome chlorosis. All samples were harvested and analyzed in three independent biological replicates.

**Figure 7:**
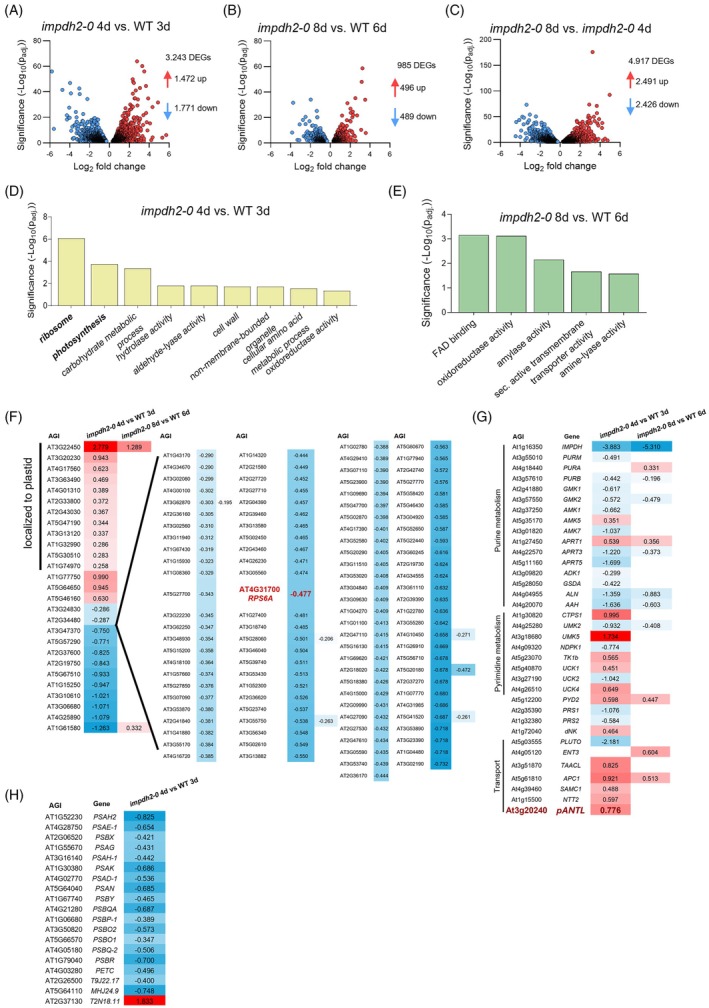
Transcriptome analysis of *impdh2‐0* and corresponding WT seedlings at two developmental stages. (A–C) Volcano plots of DEGs (*impdh2‐0* vs WT) and seedlings at same developmental stages, during their early chlorotic phase (4 days for *impdh2‐0*) and the compensated phase (8 days for *impdh2‐0*). (D, E) GO‐term analysis of transcriptomic differences measured between *impdh2‐0* and WT. (F–H) Heat map analysis of transcriptomic differences measured between *impdh2‐0* and WT seedlings of DEGs from the GO‐terms: Ribosome, Photosynthesis and Nucleotide metabolism. Numbers indicate log_2_‐fold differences.

In the comparison, *impdh2‐0 4d* versus WT 3d, 3.243 differently expressed Genes (DEGs; adjusted *P*_adj_.‐value <0.05) were identified. Among these, 1.472 were upregulated and 1.771 downregulated (Figure 7A). When comparing *impdh2‐0 8d* versus WT 6 days, only 985 DEGs were identified; app. half of them were up‐ and the other half downregulated (Figure 7B). Between 8 versus 4 days post germination of *impdh2‐0*, 4917 DEGs occurred, 2491 down and 2426 upregulated (Figure 7C). GO‐term analysis revealed that ribosome, translation, photosynthesis, and carbohydrate metabolic process are the most significantly altered GO‐Terms in 4‐day‐old *impdh2‐0* seedlings. When comparing 8‐day‐old *impdh2‐0* seedlings to WT of a comparable developmental stage, all three named categories do not appear as significant GO‐terms anymore (Figure 7D,E). Within the category ‘ribosome’ 157 altered ribosomal genes were identified in our analysis; of these, 17 genes were up‐ and 140 were down regulated (Figure 7F). Among the upregulated ribosomal proteins, 11 were chloroplast located, including AT3G22450 (log2fold change 2.78), which encodes ribosomal protein uL18‐L1. This protein is required for proper splicing of mitochondrial introns, and loss‐of‐function mutants show a dwarf phenotype (Wang et al., 2020). One of the downregulated transcripts encodes for Ribosomal Protein S6A (RPS6A), which is phosphorylated by S6K. Prior, S6K is phosphorylated by TOR (Kim et al., 2014). RPS6A is required for proper translation and may affect, in addition, histone deacetylation (Kim et al., 2014).

When inspecting ‘nucleotide metabolism’ related genes in the comparison *impdh2‐0* 4d versus WT 3d, most DEGs in purine metabolism were down, with the exception of those encoding chloroplast localized proteins APRT1 and AMK5 catalyzing the salvage of purine nucleobases (Witte & Herde, 2020) (Figure 7G). In pyrimidine metabolism, an opposite trend was observed as many genes in this category were upregulated. Uridine monophosphate kinase 5 (UMK5) represents the most strongly upregulated gene (1.7 log2 fold). Besides acting as a kinase, UMK5 (PUMPKIN) can serve as an RNA binding protein affecting plastid translation and splicing (Schmid et al., 2019). Interestingly, several organellar transporters were also upregulated, possibly to support nucleotide and energy balancing between compartments (Figure 7G). Eight‐day‐old *impdh2‐0* plants clearly showed a more WT‐like gene expression (Figure 7G). In the category Photosynthesis, all identified DEGs showed downregulation in *impdh2‐0 4d* versus WT 3d, comprising genes which encode subunits of PSI and PSII, and the expression of all these increased again in the comparison *impdh2‐0* 8d versus WT 6d (Figure 7H).

## DISCUSSION

Arabidopsis harbors two isoforms of inosine monophosphate dehydrogenase (IMPDH) and both locate to the plant cell cytosol, as revealed by analysis of fluorophore fusion proteins transiently expressed in *Nicothiana benthamiana* (Figure 1). Moreover, in a complementation approach, IMPDH1 and 2 were shown to actively convert IMP to XMP, a key step in guanylate biosynthesis (Figure 1). Finding both IMPDH isoforms catalytically active matches with strict conservation of key residues in the substrate binding sites. As *IMPDH2* exhibits much higher expression levels compared to *IMPDH1* in almost all tissues, and knock‐out mutants of this allele showed a clear developmental delay 4 days after germination this is indicative of an important function of IMPDH2 in early Arabidopsis development. Similar inhibitory effects on plant development were observed in aspartate transcarbamoylase (ATC) mutants with decreased pyrimidine *de novo* synthesis or when plants were treated with the ATC inhibitor PALA (Bellin et al., 2021; Bellin, Garza Amaya, et al., 2023; Slocum et al., 2023). In case of ATC and IMPDH mutants, reduced and most likely limiting nucleotide amounts were detected (Bellin, Melzer, et al., 2023, Figure 7). We propose that such nucleotide limitation is fundamental for the observed developmental delay. It was suggested that nucleotide reserves are depleted around 4 days after germination (Slocum et al., 2023). According to our analysis, we assume that this point is reached already 2 days after germination as in our mutants markedly reduced levels of GMP and in tendency also XMP and GTP and other nucleotides tested were seen at this time point (Figure 6; Figure S10) before photosynthesis is fully established (Pipitone et al., 2021). The observation of decreased XMP levels but increased IMP levels, latter being the substrate of IMPDH indicated that in fact IMPDH represents the bottleneck in guanylate biosynthesis and furthermore affects adenylate and cytidylate amounts. Now the question arises about the mechanism which translates limiting nucleotides into developmental delays, going along with impaired photosynthesis, reduced chlorophyll levels and reduced biomass (Figures 3 and 4). In our transcriptome analysis ‘ribosome’ showed up as major GO‐Term due to reductions in mainly cytosolic ribosomal RNA levels (Figure 6) and reduced transcription/translation of photosynthesis related genes in plastid and cytosol were observed as consequence (Figure 4D). It is meanwhile known that nucleotide limitation represses TOR activity, which then leads to reductions in ribosomal RNA levels (Busche et al., 2021). Among ribosomal genes that were reduced in expression RPS6A was identified, which is a target protein of S6K–TOR signaling (Liu et al., 2025), underlining participation of TOR in IMPDH2‐dependent seedling development. Homozygous crosses of *impdh1‐1* with *impdh2‐0* are lethal (Table S1; Maekawa et al., 2024). Obviously, there is no bypass to the catalytic step catalyzed by IMPDH. IMPDH2 is clearly the dominant isoform with respect to its expression level, which is higher than *IMPDH1* in nearly all leaf and root tissues (PLANT SINGLE CELL BROWSER; Ma et al., 2020; Maekawa et al., 2024) and the impact of *impdh2* knock‐out mutants on early plant development.

However, IMPDH1 appears to exhibit individual roles in seed development and auxin metabolism in embryos. This is shown by seed abortion in *impdh1‐1* lines and developmental phenotypes on cotyledon number and leaf and silique growth in *IMPDH1* overexpression mutants. Latter plants exhibit altered transcript levels of auxin markers (SAUR 9, 10; WOX5, WOX8; Figure 5), pointing to altered auxin gradients across the leaf, most likely causing the observed phenotypes.

As the major isoform, IMPDH2 was further analyzed with respect to its expression. Histological staining of *IMPDH2* promotor‐reporter lines supports high expression in 4‐day‐old seedlings, which was reduced by supplementation by guanine and GMP, but increased by the IMPDH inhibitor MPA indicating a regulatory impact of guanylate availability on *IMPDH2* expression (Figure S9B).

Transcriptome analysis of *impdh2‐1* revealed that besides reduced expression of ribosomal RNA encoding genes and photosynthesis related genes, several genes related to nucleotide metabolism were altered in expression (Figure 6F–H). Whereas genes involved in purine metabolism were mostly downregulated, pyrimidine metabolic genes were mostly upregulated. Strongest upregulation (1.73 log_2_ fold change impdh2‐0 versus WT) was seen for PUMPKIN (UMK5), involved in plastid RNA binding and splicing (Schmid et al., 2019). Another strongly upregulated gene was AT3G22450 (log_2_ fold change 2.78) which encodes ribosomal protein uL18‐L1. This protein is required for proper splicing of mitochondrial introns. Presumably in both cases *impdh2* mutants attempt to counteract impaired organellar RNA processing. Transporters involved in organellar energy balancing were also found upregulated (TAACL, APC1, NTT2) likely in response to reduced energy status. One gene, At3g20240, encoding the closest homolog to pANT1 (plastidial adenine nucleotide transporter) was found upregulated (Figure 6G). It is tempting to speculate that the encoded protein functions in IMP export from plastids (Haferkamp & Schmitz‐Esser, 2012; Witte & Herde, 2020). If so, increased export of IMP to overcome the bottleneck of GMP synthesis in *impdh2* would lead to the observed IMP increase (in the cytosol) and thus remove substrate for AMP synthesis in plastids. Another possible explanation of reduced AMP levels is feedback inhibition of purine biosynthesis by IMP accumulating in plastids (Chen et al., 2022).

IMPDH and CTPS, the latter enzyme catalyzing CTP synthesis from UTP, have been shown to exhibit the capacity to form filamentous structures (known as cytoophidia or rods and ring structures) in many organisms, for example human and drosophila (Keppeke et al., 2015; Liu, 2016). When we analyzed IMPDH‐Fluorophore fusion proteins by fluorescence microscopy (Figure 1) or performed BIFC analysis (Figure 2) we sometimes observe signals that are randomly dispersed, indicating solubility of the corresponding fusion proteins, but more often the fluorescence signals accumulate in punctae or oval clusters, of up to 7 μm in diameter. However, we did not observe filamentous structures that really resemble cytoophidia or rod and ring structures known from human. What can be the reason for this? First, we believe that Arabidopsis IMPDH proteins have the capacity to form filaments, because key amino acids that were identified being important for filament formation are conserved in IMPDH1 and IMPDH2 and filamentation of IMPDH was shown in organisms from all kingdoms of life (Zhou et al., 2020). It was shown that fluorescent tags can negatively influence filament formation and most likely this happened in our experiments. Nevertheless, observed clusters could be filaments that accumulate in an unusual matter and have been described as clumps in other studies (Keppeke et al., 2023). It is also possible and more likely that we do not see filaments but smaller oligomeric structures which accumulate.

Co‐assembly of mammalian IMPDH1 and IMPDH2 into the same cytoophidium was shown (Keppeke et al., 2018) and the corresponding BiFC analysis shown here supports that this can happen also in Arabidopsis (Figure 2). Interactions between IMPDH and CTPS proteins were also supported in this work, although we cannot be sure about the structural level of this interaction, may it be at the filament or oligomer level. A function of such protein–protein interaction could be a regulatory role to balance cytidylate to guanylate levels. Both metabolite groups are lower in amount compared to adenylates and uridylates, but require upregulation in a concerted way under certain physiological conditions (Straube et al., 2021). However, if protein–protein interactions between IMPDH and CTPS can contribute to such balancing requires further experiments. In human cells, an increased accumulation of IMPDH2 dependent clusters after ATP supplementation was observed and also in our experiments with AtIPDH2 (Burrell et al., 2022). This may point to a role of IMPDH filaments in the control of metabolism. However, IMPDH filaments have been adjudicated many different functions (Guo & Liu, 2023). For a better understanding of Arabidopsis IMPDH functions and if they can assemble to proper filaments, new expression systems and constructs must be tested in future. When filament formation can be shown convincingly, assembly, disassembly and the effect of metabolites, inhibitors and mutations can be tested to better understand IMPDH functions in plants.

In conclusion, our results identify IMPDH2 as the major functional IMPDH isoform in Arabidopsis and demonstrate its crucial role in maintaining guanylate nucleotide homeostasis during early seedling development. Loss of IMPDH2 activity causes nucleotide limitation, leading to impaired ribosome biogenesis, reduced photosynthetic capacity, and delayed growth, likely through repression of TOR signaling. In contrast, IMPDH1 appears to fulfill a more specialized role in seed development and auxin‐related developmental processes. Furthermore, the observed interactions between IMPDH isoforms and CTPS proteins, together with the formation of higher‐order protein assemblies, suggest additional regulatory mechanisms linking nucleotide metabolism to plant growth and development. Future studies should clarify the structural nature and physiological significance of these assemblies and further elucidate how nucleotide availability is integrated into developmental signaling networks in plants.

## EXPERIMENTAL PROCEDURES

### Plant growth

WT and transgenic *A. thaliana* (L.) Heynh. Plants (ecotype Columbia) were used for DNA isolation, tissue collection, and phenotypic inspection throughout. Standardized ED73 soil with 10% sand (Einheitserde und Humuswerke Patzer) or ½ MS agar plates were used for plant growth under short day (SD) conditions with 10 h light (120 μmol quanta m^−2^ sec^−1^); 22°C and 60% humidity. LED lights (Valoya NS1, Valoya, Finland) were used for illumination. For growth experiments on sterile agar plates and liquid cultures, seeds were surface sterilized and placed on half‐strength MS supplemented with 0.1% (w/v) sucrose. For supplementation experiments, the medium was enriched with either 0.5 mM guanine, 0.5 mM GMP, or 10 μM mycophenolic acid (MPA). Seeds were incubated for 48 h in the dark at 4°C for imbibition (Weigel & Glazebrook, 2002). Liquid cultivation was performed in 6 well plates with 5 mL media per well and constant rotation at 100 rpm.

### Construction of IMPDH2 reporter lines, *IMPDH1* complementation lines and *IMPDH1/2* overexpression lines

Construction of promoter GUS lines by gateway cloning into the pBGWF7.0 vector was performed as described before (Hickl et al., 2021). For this, 1889 bp upstream of the start codon of At1g16350 (*IMPDH2*) was amplified from WT gDNA and inserted into pBGWF7.0. Primers used are given in Table S2. pMDC123 was used for complementation of *impdh1‐1* with the *IMPDH1* full‐length native genomic construct including the promoter as given above (Curtis & Grossniklaus, 2003). Overexpression of both *IMPDH1* and *IMPDH2* from WT cDNA was achieved by Gateway Cloning into pUBC (Grefen et al., 2010), resulting in control of transgene expression by the ubiquitin10 promoter. Correctness of the cloning procedure was checked by enzyme digestion and Sanger sequencing (Eurofins, Ebersberg). Transformation into WT plants and *IMPDH1* mutants was performed according to the floral inoculation procedure (Narusaka et al., 2010).

### Cloning of IMPDH‐CFP and ‐GFP reporter constructs and constructs for BIFC analysis

Full length genes were used for all cloning procedures described here. IMPDH‐CFP and ‐GFP constructs were obtained by gateway cloning of corresponding pDONR inserts into pUBC‐CFP or GFP destination vectors (Grefen et al., 2010). Cloning of CTPS BIFC constructs was described in (Krämer et al., 2022) and IMPDH BIFC construct were generated the same way. As negative control, dehydroorotase (DHO, At4g22930, Moffatt & Ashihara, 2002; Witz et al., 2012) was used. In short: After initial gateway cloning into pDONR, genes were introduced into pUBC‐cYFP, pUBC‐nYFP, pUBN‐cYFP, and pUBN‐nYFP vectors (Grefen et al., 2010), resulting in localization of YFP halves (nYFP or cYFP) to the N‐ or C‐terminus of corresponding genes. These constructs were then transformed into *A. tumefaciens* strain GV3101 as given below.

### Transcript quantification with qRT PCR

The isolation of RNA, subsequent preparation of cDNA, and PCR were conducted in accordance with the methods outlined in Ohler et al. (2019). Leaf tissue was frozen using liquid nitrogen, followed by grinding to a fine powder. Subsequently, RNA was isolated using the Nucleospin RNA Plant Kit (Macherey‐Nagel, Düren, Germany) in accordance with the manufacturer's instructions. The isolated RNA was then transcribed into cDNA using the qScript cDNA Synthesis Kit (Quantabio, United States). The Primers used are listed in Table S2.

### Transcriptome analysis

A comprehensive expression analysis of WT and impdh2 knock‐out plants was performed to obtain an overview of the transcriptome during early seedling development. For this purpose, WT, *impdh2‐0* and *ami‐ctps2‐2* seedlings at the same stage of development were harvested in the chlorotic growth phase and in the greening phase. Two hours after the start of the light phase, the seedlings without roots were harvested and transferred directly to liquid nitrogen. Isolated RNA samples were sent to Novogene (China), which created the RNA library and performed transcriptome sequencing. The online tool REVIGO (Supek et al., 2011) was used to evaluate the GO‐term analysis.

### Protein extraction, gel‐electrophoresis and immunoblotting

50–100 mg leaf samples were grinded in liquid nitrogen and subsequently extracted with protein extraction buffer (50 mM Hepes‐KOH, pH 7.8, 5 mM MgCl, 1 mM ethylenediaminetetraacetic acid, 2 mM dithiothreitol, 0.01% DDM and 2% (v/v) protease inhibitor cocktail (Sigma, St Louis, MO)). Supernatants were then centrifuged at 13 000 ***g*** for 1 min at 4°C. Protein extracts were supplemented with 4× Laemmli buffer and the samples were stored at −80°C until use. SDS PAGE was performed with precast SDS gels (BioRad, Hercules, CA, USA) according to the manufacturer's protocol.

Immunoblots were essentially performed as given in Ohler et al. (2019), here the proteins were separated on SDS gels and transferred onto a nitrocellulose membrane by the semi‐dry Transblot Turbo Transfer System (BioRad, Hercules, CA, USA). Primary antibodies were obtained from Agrisera (Vännäs, Sweden).

### Photosynthetic activity was measured using pulse amplitude modulation (PAM)

Chlorophyll fluorescence was measured using a MINI Imaging pulse amplitude modulation fluorometer (Walz, www.walz.com). Prior to measurement, plants were adapted to the dark for 10 min. The quantum yield of photochemical energy conversion on PSII Φ_(PSII)_ and the quantum yield of non‐photochemical quenching of chlorophyll fluorescence Φ_(NPQ)_ and unregulated energy dissipation Φ_(NO)_ were determined (Kramer et al., 2004). Induction curves were run for 5.4 min and actinic light (76 μmol photons m^−2^ sec^−1^) provided from 40 sec time point onward, every 20 sec.

### Statistical analysis

Statistical analyses of numerical data were performed using GraphPad Prism 10. To test for significant differences between two groups, a one‐way ANOVA test followed by a Dunnett multiple comparison test was performed (*P‐value < 0.05; **P‐value < 0.01, ***P‐value <0.001, ****P‐value < 0.0001). The ‘Rapid Publication‐Ready MS Word Tables Using Two‐Way ANOVA’ software (Assaad et al., 2015) (https://houssein‐assaad.shinyapps.io/TwoWayANOVA/) was used for a letter‐based representation of the pairwise comparisons, using a two‐sided ANOVA followed by *post hoc* Tukey HSD testing.

### Transient expression in *N. benthamiana* and microscopy

Transient expression of IMPDH fused to YFP, GFP, or CFP was performed as detailed in Walter et al. (2004). Therefore, 6 weeks old *N. benthamiana* leaves were infiltrated through the lower epidermis. After 4–5 days, leaves were analyzed for the presence of fluorescence signals with a Leica TCS SP5II microscope (CFP 355 nm excitation and 495–530 nm detection, YFP 514 nm excitation and 525–582 nm detection, GFP 488 nm excitation and 505–540 nm detection of emission through a HCX PL APO 63 × 1.2 W water immersion objective). Chlorophyll autofluorescence was detected with 514 nm excitation and a 651–704 nm emission wavelength.

### Chlorophyll quantification

Leaf tissue was flash frozen in liquid nitrogen and subsequently ground to a fine powder. Subsequently, 80% ethanol was added, and the sample was subjected to heating at a temperature of 95°C for 10 min followed by sedimentation of the insoluble contents by centrifugation at 20 000 ***g*** for 10 min. The chlorophyll content of the sample was determined by measuring its absorbance at 652 nm and calculating Chlorophyll A and B (Porra & Scheer, 2019).

### Lipid extraction

Seeds (100 mg) were ground in liquid nitrogen and lipids were extracted by addition of 750 μl isopropanol under constant shaking for 12 h at 4°C (Reiser et al., 2004). Lipid extracts were then dried and dissolved in chloroform and subsequently purified using solid‐phase extraction (SPE). In the thin‐layer chromatography (TLC) procedure, lipid extract and standards (rice oil or triacylglycerol) were applied to silica gel plates and developed in hexane diethyl ether acetic acid (70:30:1). The presence of lipid bands was determined with iodine staining.

### Nucleotide quantification

For cultivation of 0, 12 and 48 h seed and seedling samples a previously described protocol was used (Niehaus et al., 2022). Eight‐day‐old seedlings were grown on soil as described above. Samples for 0 and 12 h were lyophilized prior to nucleotide extraction to remove solid frozen liquid interfering with sample grinding. Nucleotide quantification was then performed as described in Straube et al. (2021) with modifications from Straube et al. (2023).

## CONFLICT OF INTEREST

No conflicts of interest declared.

## Supporting information


**Figure S1.** Phylogenetic tree of IMPDH isoforms across the kingdoms of life.

**Figure S2.** Alignment of selected IMPDH proteins.

**Figure S3.** Genotyping *Escherichia coli IMPDH* complementation.

**Figure S4.** Representative images of IMPDH‐fluorophore fusions.

**Figure S5.** BiFC analysis of *IMPDH* and *CTPS* isoforms.

**Figure S6.** Metabolite dependent polymer formation in *IMPDH2:GFP*.

**Figure S7.** Genotyping of *IMPDH1* and *IMPDH2* t‐DNA Insertion, complementation and overexpression lines.

**Figure S8.** Photosynthetic alterations in *IMPDH* mutants.

**Figure S9.** Supplementation of *IMPDH2* mutants.

**Figure S10.** Nucleotide levels in *IMPDH* mutants.

**Table S1.** Genotyping of het. *impdh1‐1* x *impdh2‐0* offspring.

**Table S2.** Primers used in this study.

## Data Availability

The data discussed in this publication have been deposited in NCBI's Gene Expression Omnibus (Edgar et al., 2002) and are accessible through GEO Series accession number GSE312534 (https://www.ncbi.nlm.nih.gov/geo/query/acc.cgi?acc=GSE268410).

## References

[tpj71072-bib-0001] Alamdari, K. , Fisher, K.E. , Tano, D.W. , Rai, S. , Palos, K. , Nelson, A.D.L. et al. (2021) Chloroplast quality control pathways are dependent on plastid DNA synthesis and nucleotides provided by cytidine triphosphate synthase two. The New Phytologist, 231, 1431–1448. 33993494 10.1111/nph.17467

[tpj71072-bib-0002] Anthony, S.A. , Burrell, A.L. , Johnson, M.C. , Duong‐Ly, K.C. , Kuo, Y.M. , Simonet, J.C. et al. (2017) Reconstituted IMPDH polymers accommodate both catalytically active and inactive conformations. Molecular Biology of the Cell, 28, 2600–2608. 28794265 10.1091/mbc.E17-04-0263 PMC5620369

[tpj71072-bib-0003] Assaad, H.I. , Hou, Y. , Zhou, L. , Carroll, R.J. & Wu, G. (2015) Rapid publication‐ready MS‐Word tables for two‐way ANOVA. Springerplus, 4, 33. 25635246 10.1186/s40064-015-0795-z PMC4305362

[tpj71072-bib-0004] Bellin, L. , Del Cano‐Ochoa, F. , Velazquez‐Campoy, A. , Möhlmann, T. & Ramon‐Maiques, S. (2021) Mechanisms of feedback inhibition and sequential firing of active sites in plant aspartate transcarbamoylase. Nature Communications, 12, 947. 10.1038/s41467-021-21165-9 PMC7878868 33574254

[tpj71072-bib-0005] Bellin, L. , Garza Amaya, D.L. , Scherer, V. , Pruss, T. , John, A. , Richter, A. et al. (2023) Nucleotide imbalance, provoked by downregulation of aspartate transcarbamoylase impairs cold acclimation in Arabidopsis. Molecules, 28, 1585. 36838573 10.3390/molecules28041585 PMC9959217

[tpj71072-bib-0006] Bellin, L. , Melzer, M. , Hilo, A. , Garza Amaya, D.L. , Keller, I. , Meurer, J. et al. (2023) Nucleotide limitation results in impaired photosynthesis, reduced growth and seed yield together with massively altered gene expression. Plant & Cell Physiology, 64, 1494–1510. 37329302 10.1093/pcp/pcad063

[tpj71072-bib-0007] Bowne, S.J. , Sullivan, L.S. , Blanton, S.H. , Cepko, C.L. , Blackshaw, S. , Birch, D.G. et al. (2002) Mutations in the inosine monophosphate dehydrogenase 1 gene (IMPDH1) cause the RP10 form of autosomal dominant retinitis pigmentosa. Human Molecular Genetics, 11, 559–568. 11875050 10.1093/hmg/11.5.559 PMC2585828

[tpj71072-bib-0008] Buey, R.M. , Ledesma‐Amaro, R. , Velazquez‐Campoy, A. , Balsera, M. , Chagoyen, M. , de Pereda, J.M. et al. (2015) Guanine nucleotide binding to the Bateman domain mediates the allosteric inhibition of eukaryotic IMP dehydrogenases. Nature Communications, 6, 8923. 10.1038/ncomms9923 PMC4660370 26558346

[tpj71072-bib-0009] Burrell, A.L. & Kollman, J.M. (2022) IMPDH dysregulation in disease: a mini review. Biochemical Society Transactions, 50, 71–82. 35191957 10.1042/BST20210446 PMC9022972

[tpj71072-bib-0010] Burrell, A.L. , Nie, C. , Said, M. , Simonet, J.C. , Fernandez‐Justel, D. , Johnson, M.C. et al. (2022) IMPDH1 retinal variants control filament architecture to tune allosteric regulation. Nature Structural & Molecular Biology, 29, 47–58. 10.1038/s41594-021-00706-2 PMC9044917 35013599

[tpj71072-bib-0011] Busche, M. , Scarpin, M.R. , Hnasko, R. & Brunkard, J.O. (2021) TOR coordinates nucleotide availability with ribosome biogenesis in plants. Plant Cell, 33, 1615–1632. Available from: 10.1093/plcell/koab043 33793860 PMC8254494

[tpj71072-bib-0012] Cao, Y. & Schubert, K.R. (2001) Molecular cloning and characterization of a cDNA encoding soybean nodule IMP dehydrogenase. Biochimica et Biophysica Acta, 1520, 242–246. 11566360 10.1016/s0167-4781(01)00269-x

[tpj71072-bib-0013] Carr, S.F. , Papp, E. , Wu, J.C. & Natsumeda, Y. (1993) Characterization of human type I and type II IMP dehydrogenases. The Journal of Biological Chemistry, 268, 27286–27290. 7903306

[tpj71072-bib-0014] Chang, C.C. , Keppeke, G.D. , Sung, L.Y. & Liu, J.L. (2018) Interfilament interaction between IMPDH and CTPS cytoophidia. The FEBS Journal, 285, 3753–3768. 30085408 10.1111/febs.14624 PMC6220823

[tpj71072-bib-0015] Chang, Y.F. & Carman, G.M. (2008) CTP synthetase and its role in phospholipid synthesis in the yeast *Saccharomyces cerevisiae* . Progress in Lipid Research, 47, 333–339. 18439916 10.1016/j.plipres.2008.03.004 PMC2583782

[tpj71072-bib-0016] Chen, X. , Kim, S.H. , Rhee, S. & Witte, C.P. (2022) A plastid nucleoside kinase is involved in inosine salvage and control of purine nucleotide biosynthesis. Plant Cell, 35, 510–528. Available from: 10.1093/plcell/koac320 PMC9806653 36342213

[tpj71072-bib-0017] Collart, F.R. , Osipiuk, J. , Trent, J. , Olsen, G.J. & Huberman, E. (1996) Cloning and characterization of the gene encoding IMP dehydrogenase from *Arabidopsis thaliana* . Gene, 174, 217–220. 8890737 10.1016/0378-1119(96)00045-5

[tpj71072-bib-0018] Curtis, M.D. & Grossniklaus, U. (2003) A gateway cloning vector set for high‐throughput functional analysis of genes in planta. Plant Physiology, 133, 462–469. 14555774 10.1104/pp.103.027979 PMC523872

[tpj71072-bib-0019] Daumann, M. , Hickl, D. , Zimmer, D. , DeTar, R.A. , Kunz, H.H. & Möhlmann, T. (2018) Characterization of filament‐forming CTP synthases from *Arabidopsis thaliana* . The Plant Journal, 96, 316–328. 30030857 10.1111/tpj.14032 PMC6821390

[tpj71072-bib-0020] Edgar, R. , Domrachev, M. & Lash, A.E. (2002) Gene expression omnibus: NCBI gene expression and hybridization array data repository. Nucleic Acids Research, 30, 207–210. 11752295 10.1093/nar/30.1.207 PMC99122

[tpj71072-bib-0061] Furutani, M. , Vernoux, T. , Traas, J. , Kato, T. , Tasaka, M. & Aida, M. (2004) PIN‐FORMED1 and PINOID regulate boundary formation and cotyledon development in Arabidopsis embryogenesis. Development, 131, 5021–5030. 15371311 10.1242/dev.01388

[tpj71072-bib-0021] Grefen, C. , Donald, N. , Hashimoto, K. , Kudla, J. , Schumacher, K. & Blatt, M.R. (2010) A ubiquitin‐10 promoter‐based vector set for fluorescent protein tagging facilitates temporal stability and native protein distribution in transient and stable expression studies. The Plant Journal, 64, 355–365. 20735773 10.1111/j.1365-313X.2010.04322.x

[tpj71072-bib-0022] Guo, C.J. & Liu, J.L. (2023) Cytoophidia and filaments: you must unlearn what you have learned. Biochemical Society Transactions, 51, 1245–1256. 37248970 10.1042/BST20221410

[tpj71072-bib-0023] Haferkamp, I. & Schmitz‐Esser, S. (2012) The plant mitochondrial carrier family: functional and evolutionary aspects. Frontiers in Plant Science, 3, 2. 22639632 10.3389/fpls.2012.00002 PMC3355725

[tpj71072-bib-0062] Hamann, T. , Benkova, E. , Bäurle, I. , Kientz, M. & Jürgens, G. (2002) The Arabidopsis BODENLOS gene encodes an auxin response protein inhibiting MONOPTEROS‐mediated embryo patterning. Genes & Development, 16, 1610–1615. 12101120 10.1101/gad.229402 PMC186366

[tpj71072-bib-0024] Hedstrom, L. (2009) IMP dehydrogenase: structure, mechanism, and inhibition. Chemical Reviews, 109, 2903–2928. 19480389 10.1021/cr900021w PMC2737513

[tpj71072-bib-0025] Hickl, D. , Scheuring, D. & Möhlmann, T. (2021) CTP synthase 2 from *Arabidopsis thaliana* is required for complete embryo development. Frontiers in Plant Science, 12, 652434. 33936137 10.3389/fpls.2021.652434 PMC8082242

[tpj71072-bib-0026] Hölzl, G. & Dörmann, P. (2019) Chloroplast lipids and their biosynthesis. Annual Review of Plant Biology, 70, 51–81. 10.1146/annurev-arplant-050718-100202 30786236

[tpj71072-bib-0027] Jackson, R.C. , Weber, G. & Morris, H.P. (1975) IMP dehydrogenase, an enzyme linked with proliferation and malignancy. Nature, 256, 331–333. 167289 10.1038/256331a0

[tpj71072-bib-0028] Johnson, M.C. & Kollman, J.M. (2020) Cryo‐EM structures demonstrate human IMPDH2 filament assembly tunes allosteric regulation. eLife, 9, e53243. 31999252 10.7554/eLife.53243 PMC7018514

[tpj71072-bib-0029] Keppeke, G.D. , Calise, S.J. , Chan, E.K. & Andrade, L.E. (2015) Assembly of IMPDH2‐based, CTPS‐based, and mixed rod/ring structures is dependent on cell type and conditions of induction. Journal of Genetics and Genomics, 42, 287–299. 26165495 10.1016/j.jgg.2015.04.002

[tpj71072-bib-0030] Keppeke, G.D. , Chang, C.C. , Peng, M. , Chen, L.Y. , Lin, W.C. , Pai, L.M. et al. (2018) IMP/GTP balance modulates cytoophidium assembly and IMPDH activity. Cell Div, 13, 5. 29946345 10.1186/s13008-018-0038-0 PMC6004095

[tpj71072-bib-0031] Keppeke, G.D. , Chang, C.C. , Zhang, Z. & Liu, J.L. (2023) Effect on cell survival and cytoophidium assembly of the adRP‐10‐related IMPDH1 missense mutation Asp226Asn. Frontiers in Cell and Developmental Biology, 11, 1234592. 37731818 10.3389/fcell.2023.1234592 PMC10507268

[tpj71072-bib-0032] Kim, Y.K. , Kim, S. , Shin, Y.J. , Hur, Y.S. , Kim, W.Y. , Lee, M.S. et al. (2014) Ribosomal protein S6, a target of rapamycin, is involved in the regulation of rRNA genes by possible epigenetic changes in Arabidopsis. Journal of Biological Chemistry, 289, 3901–3912. 24302738 10.1074/jbc.M113.515015 PMC3924259

[tpj71072-bib-0033] Kramer, D.M. , Johnson, G. , Kiirats, O. & Edwards, G.E. (2004) New fluorescence parameters for the determination of QA redox state and excitation energy fluxes. Photosynthesis Research, 79, 209–218. 16228395 10.1023/B:PRES.0000015391.99477.0d

[tpj71072-bib-0034] Krämer, M. , Dörfer, E. , Hickl, D. , Bellin, L. , Scherer, V. & Möhlmann, T. (2022) Cytidine triphosphate synthase four from *Arabidopsis thaliana* attenuates drought stress effects. Frontiers in Plant Science, 13, 842156. 35360303 10.3389/fpls.2022.842156 PMC8960734

[tpj71072-bib-0035] Leroch, M. , Kirchberger, S. , Haferkamp, I. , Wahl, M. , Neuhaus, H.E. & Tjaden, J. (2005) Identification and characterization of a novel plastidic adenine nucleotide uniporter from *Solanum tuberosum* . The Journal of Biological Chemistry, 280, 17992–18000. 15737999 10.1074/jbc.M412462200

[tpj71072-bib-0036] Levitzki, A. & Koshland, D.E., Jr. (1972) Role of an allosteric effector. Guanosine triphosphate activation in cytosine triphosphate synthetase. Biochemistry, 11, 241–246. 4550559 10.1021/bi00752a015

[tpj71072-bib-0037] Liu, J.L. (2016) The cytoophidium and its kind: filamentation and compartmentation of metabolic enzymes. Annual Review of Cell and Developmental Biology, 32, 349–372. 10.1146/annurev-cellbio-111315-124907 27362644

[tpj71072-bib-0038] Liu, Y. , Hu, J. , Duan, X. , Ding, W. , Xu, M. & Xiong, Y. (2025) Target of rapamycin (TOR): a master regulator in plant growth, development, and stress responses. Annual Review of Plant Biology, 76, 341–371. 10.1146/annurev-arplant-083123-050311 39952681

[tpj71072-bib-0039] Lunn, F.A. , MacDonnell, J.E. & Bearne, S.L. (2008) Structural requirements for the activation of *Escherichia coli* CTP synthase by the allosteric effector GTP are stringent, but requirements for inhibition are lax. The Journal of Biological Chemistry, 283, 2010–2020. 18003612 10.1074/jbc.M707803200

[tpj71072-bib-0063] Ma, X. , Denyer, T. & Timmermans, M.C. (2020) PscB: a browser to explore plant single cell RNA‐sequencing data sets. Plant Physiology, 183, 464–467. 32209591 10.1104/pp.20.00250 PMC7271789

[tpj71072-bib-0040] Maekawa, S. , Nishikawa, I. & Horiguchi, G. (2024) Impaired inosine monophosphate dehydrogenase leads to plant‐specific ribosomal stress responses in *Arabidopsis thaliana* . Journal of Plant Research, 137, 1091–1104. 39235732 10.1007/s10265-024-01578-5

[tpj71072-bib-0041] Maraschin Fdos, S. , Memelink, J. & Offringa, R. (2009) Auxin‐induced, SCF(TIR1)‐mediated poly‐ubiquitination marks AUX/IAA proteins for degradation. The Plant Journal, 59, 100–109. 19309453 10.1111/j.1365-313X.2009.03854.x

[tpj71072-bib-0042] Moffatt, B.A. & Ashihara, H. (2002) Purine and pyrimidine nucleotide synthesis and metabolism. The Arabidopsis Book, 4(1), e0018. Available from: 10.1199/tab.0018 PMC3243375 22303196

[tpj71072-bib-0043] Narusaka, M. , Shiraishi, T. , Iwabuchi, M. & Narusaka, Y. (2010) The floral inoculating protocol: a simplified *Arabidopsis thaliana* transformation method modified from floral dipping. Plant Biotechnology, 27, 349–351.

[tpj71072-bib-0044] Niehaus, M. , Straube, H. , Specht, A. , Baccolini, C. , Witte, C.P. & Herde, M. (2022) The nucleotide metabolome of germinating *Arabidopsis thaliana* seeds reveals a central role for thymidine phosphorylation in chloroplast development. Plant Cell, 34, 3790–3813. 35861422 10.1093/plcell/koac207 PMC9516053

[tpj71072-bib-0045] Ohler, L. , Niopek‐Witz, S. , Mainguet, S.E. & Möhlmann, T. (2019) Pyrimidine salvage: physiological functions and interaction with chloroplast biogenesis. Plant Physiology, 180, 1816–1828. 31101721 10.1104/pp.19.00329 PMC6670073

[tpj71072-bib-0046] Pipitone, R. , Eicke, S. , Pfister, B. , Glauser, G. , Falconet, D. , Uwizeye, C. et al. (2021) A multifaceted analysis reveals two distinct phases of chloroplast biogenesis during de‐etiolation in Arabidopsis. eLife, 10, e62709. 33629953 10.7554/eLife.62709 PMC7906606

[tpj71072-bib-0047] Porra, R.J. & Scheer, H. (2019) Towards a more accurate future for chlorophyll a and b determinations: the inaccuracies of Daniel Arnon's assay. Photosynthesis Research, 140, 215–219. 30194670 10.1007/s11120-018-0579-8

[tpj71072-bib-0048] Reiser, J. , Linka, N. , Lemke, L. , Jeblick, W. & Neuhaus, H.E. (2004) Molecular physiological analysis of the two plastidic ATP/ADP transporters from Arabidopsis. Plant Physiology, 136, 3524–3536. 15516503 10.1104/pp.104.049502 PMC527152

[tpj71072-bib-0049] Schmid, L.M. , Ohler, L. , Möhlmann, T. , Brachmann, A. , Muino, J.M. , Leister, D. et al. (2019) PUMPKIN, the sole plastid UMP kinase, associates with group II introns and alters their metabolism. Plant Physiology, 179, 248–264. 30409856 10.1104/pp.18.00687 PMC6324238

[tpj71072-bib-0050] Sifri, C.D. , Wilson, K. , Smolik, S. , Forte, M. & Ullman, B. (1994) Cloning and sequence analysis of a *Drosophila melanogaster* cDNA encoding IMP dehydrogenase. Biochimica et Biophysica Acta, 1217, 103–106. 7904480

[tpj71072-bib-0051] Slocum, R.D. , Peña, C.M. & Liu, Z.C. (2023) Transcriptional reprogramming of nucleotide metabolism in response to altered pyrimidine availability in Arabidopsis seedlings. Frontiers in Plant Science, 14, 1273235. 38023851 10.3389/fpls.2023.1273235 PMC10652772

[tpj71072-bib-0052] Straube, H. , Niehaus, M. , Zwittian, S. , Witte, C.P. & Herde, M. (2021) Enhanced nucleotide analysis enables the quantification of deoxynucleotides in plants and algae revealing connections between nucleoside and deoxynucleoside metabolism. Plant Cell, 33, 270–289. 33793855 10.1093/plcell/koaa028 PMC8136904

[tpj71072-bib-0053] Straube, H. , Straube, J. , Rinne, J. , Fischer, L. , Niehaus, M. , Witte, C.P. et al. (2023) An inosine triphosphate pyrophosphatase safeguards plant nucleic acids from aberrant purine nucleotides. The New Phytologist, 237, 1759–1775. 36464781 10.1111/nph.18656

[tpj71072-bib-0054] Supek, F. , Bosnjak, M. , Skunca, N. & Smuc, T. (2011) REVIGO summarizes and visualizes long lists of gene ontology terms. PLoS One, 6, e21800. 21789182 10.1371/journal.pone.0021800 PMC3138752

[tpj71072-bib-0064] Walter, M. , Chaban, C. , Schütze, K. , Batistic, O. , Weckermann, K. , Näke, C. et al. (2004) Visualization of protein interactions in living plant cells using bimolecular fluorescence complementation. The Plant Journal, 40, 428–438. 15469500 10.1111/j.1365-313X.2004.02219.x

[tpj71072-bib-0055] Wang, C. , Fourdin, R. , Quadrado, M. , Dargel‐Graffin, C. , Tolleter, D. , Macherel, D. et al. (2020) Rerouting of ribosomal proteins into splicing in plant organelles. Proceedings of the National Academy of Sciences of the United States of America, 117, 29979–29987. 33168708 10.1073/pnas.2004075117 PMC7703591

[tpj71072-bib-0056] Weigel, D. & Glazebrook, J. (2002) Arabidopsis. A Laboratory Manual. New York: Cold Spring Harbor Laboratory Press.

[tpj71072-bib-0057] Witte, C.P. & Herde, M. (2020) Nucleotide metabolism in plants. Plant Physiology, 182, 63–78. 31641078 10.1104/pp.19.00955 PMC6945853

[tpj71072-bib-0058] Witz, S. , Jung, B. , Fürst, S. & Möhlmann, T. (2012) De novo pyrimidine nucleotide synthesis mainly occurs outside of plastids, but a previously undiscovered nucleobase importer provides substrates for the essential salvage pathway in Arabidopsis. Plant Cell, 24, 1549–1559. 22474184 10.1105/tpc.112.096743 PMC3398563

[tpj71072-bib-0059] Zhou, S. , Xiang, H. & Liu, J.L. (2020) CTP synthase forms cytoophidia in archaea. Journal of Genetics and Genomics, 47, 213–223. 32507415 10.1016/j.jgg.2020.03.004

[tpj71072-bib-0060] Zrenner, R. , Stitt, M. , Sonnewald, U. & Boldt, R. (2006) Pyrimidine and purine biosynthesis and degradation in plants. Annual Review of Plant Biology, 57, 805–836. 10.1146/annurev.arplant.57.032905.105421 16669783

